# Uncovering the effect of low-frequency static magnetic field on tendon-derived cells: from mechanosensing to tenogenesis

**DOI:** 10.1038/s41598-017-11253-6

**Published:** 2017-09-08

**Authors:** Tamagno Pesqueira, Raquel Costa-Almeida, Manuela E. Gomes

**Affiliations:** 1 3B’s Research Group – Biomaterials, Biodegradables and Biomimetics, University of Minho, Headquarters of the European Institute of Excellence on Tissue Engineering and Regenerative Medicine, Avepark – Parque de Ciência e Tecnologia, Zona Industrial da Gandra, 4805-017 Barco, Guimarães Portugal; 2 ICVS/3B’s – PT Government Associate Laboratory, Guimarães, Portugal

## Abstract

Magnetotherapy has been receiving increased attention as an attractive strategy for modulating cell physiology directly at the site of injury, thereby providing the medical community with a safe and non-invasive therapy. Yet, how magnetic field influences tendon cells both at the cellular and molecular levels remains unclear. Thus, the influence of a low-frequency static magnetic field (2 Hz, 350 mT) on human tendon-derived cells was studied using different exposure times (4 and 8 h; short-term studies) and different regimens of exposure to an 8h-period of magnetic stimulation (continuous, every 24 h or every 48 h; long-term studies). Herein, 8 h stimulation in short-term studies significantly upregulated the expression of tendon-associated genes *SCX*, C*OL1A1*, *TNC* and *DCN* (*p* < 0.05) and altered intracellular Ca^2+^ levels (*p* < 0.05). Additionally, every 24 h regimen of stimulation significantly upregulated *COL1A1*, *COL3A1* and *TNC* at day 14 in comparison to control (p < 0.05), whereas continuous exposure differentially regulated the release of the immunomodulatory cytokines IL-1β and IL-10 (p < 0.001) but only at day 7 in comparison to controls. Altogether, these results provide new insights on how low-frequency static magnetic field fine-tune the behaviour of tendon cells according to the magnetic settings used, which we foresee to represent an interesting candidate to guide tendon regeneration.

## Introduction

Tendons are mechanoresponsive tissues that enable the communication of mechanical forces generated by skeletal muscles to bones; hence, they are constantly subjected to mechanical forces *in vivo* during daily activities. Therefore, the understanding of tendon biology encompasses the effects of mechanical forces on balancing between tissue homeostasis and the development of pathologies. In this regard, mechanical stimulation is of particular relevance when addressing tendon regeneration strategies or envisioning *in vitro* emulation of tendon niche. For example, mechanically stimulated (uniaxial cyclic stretching 0.5 Hz, 4% strain) tendon stem cells increased the gene expression of scleraxis^1^, a transcription factor expressed in both tendon stem/progenitor cells and mature tendon tissue^2^, which is also involved in the regulation of a latter differentiation marker of tenocytes, tenomodulin^3^. Additionally, an upregulation in the expression of collagen type I and tenascin C was also observed at the gene level upon mechanical loading on a three-dimensional (3D) environment, immediately after exposure to uniaxial cyclic stretching^1, 4^.

Over the years, magnetic stimulation and magnetically actuated biomaterials have been receiving increased attention toward the establishment of novel therapies aiming at tissue regeneration^5, 6^. Magnetic forces correspond to a subcategory of physical forces, which are of utmost importance in governing cellular processes, such as proliferation and differentiation, as well as gene expression and secretion of extracellular matrix (ECM) proteins^7^. Clinically, distinct magnetotherapy modalities have been approved by US Food and Drug Administration (FDA) for orthopaedic applications, including biphasic low-frequency magnetic field for non-union fractures, as well as pulsed radiofrequency electromagnetic field for treating pain and edema in superficial soft tissues^8^. For instance, exposure to magnetic field has been reported to enhance cartilage and bone repair through increased matrix formation^9–11^. In addition, the application of a combined magnetic field (dynamic sinusoidal magnetic field and a magnetostatic field) to a rabbit model of partial patellectomy resulted in enhanced healing at the tendon-to-bone junction, achieved through the formation of new bone tissue, regeneration of the fibrocartilage zone and improved mechanical properties^12^.

In the particular case of tendons, electromagnetic actuation has been applied to patients suffering from persistent rotator cuff tendinitis and has shown positive effects in aiding tissue repair by reducing pain symptoms and improving the range of active movement^13^. Moreover, *in vivo* application of pulsed electromagnetic field in a rat model of Achilles tendon transection resulted in a 69% increase of tensile strength at the repair site, in comparison to non-stimulated controls^14^. Additionally, the application of an external magnetic field can be explored toward enhancing the maturation of tissue engineered constructs *in vitro* prior to implantation. Thus, we have been exploring the use of lower frequencies to understand the potential role of these magnetic field settings as a mechanical stimulus on human cells^15–17^. For instance, in a previous work, we have demonstrated that the application of a low-frequency magnetic field promoted tenogenic differentiation of human adipose stem cells cultured on aligned magnetic scaffolds by enhancing the deposition of tendon-like ECM (collagen type I and tenascin C)^5^. Despite such satisfactory outcomes, understanding the behaviour of resident tendon cells by tracking the molecular changes is vital to enhance tissue regeneration, ultimately providing new insights into the applications of magnetotherapy in orthopaedics, either by contact-free direct application on injured tendons or as a mechano-magnetic stimulus in the development of tissue engineered constructs.

In this work, we aimed at exploring the effects of low-frequency magnetic field as an alternative to electromagnetic fields already in use in modulating the physiology of tendon-derived cells, not only at tendon gene and protein levels but possible mechanosensing apparatus responsible for converting magnetic signals into the biological response. In particular, the hypothesis underlying herein is that the application of a magnetic field can be perceived by tendon cells as a mechanical stimulus, and different exposure times and regimens of stimulation lead to alterations at gene and protein expression level. Thus, we exposed human tendon-derived cells (hTDCs) to different periods of magnetic stimulation and demonstrated that both 4 h and 8 h magnetic stimulation (herein called short-term studies) altered intracellular calcium dynamics but had no effects on the expression of vinculin and focal adhesion kinase. On the other hand, 8 h stimulation resulted in an increased gene expression of scleraxis, collagen type I, tenascin C and decorin. Moreover, exposing hTDCs every 24 h upregulated the expression of collagen types I and III, and tenascin C after 14 days of magnetic stimulation, in comparison to unexposed cells. Furthermore, continuous stimulation decreased the release of pro-inflammatory interleukin (IL)-1β but increased anti-inflammatory IL-10 in comparison to unexposed cells at day 7 of culture.

## Results

### Exposure to low-frequency static magnetic field altered intracellular calcium dynamics and the production of reactive oxygen species in a time-dependent manner

**Figure 1:**
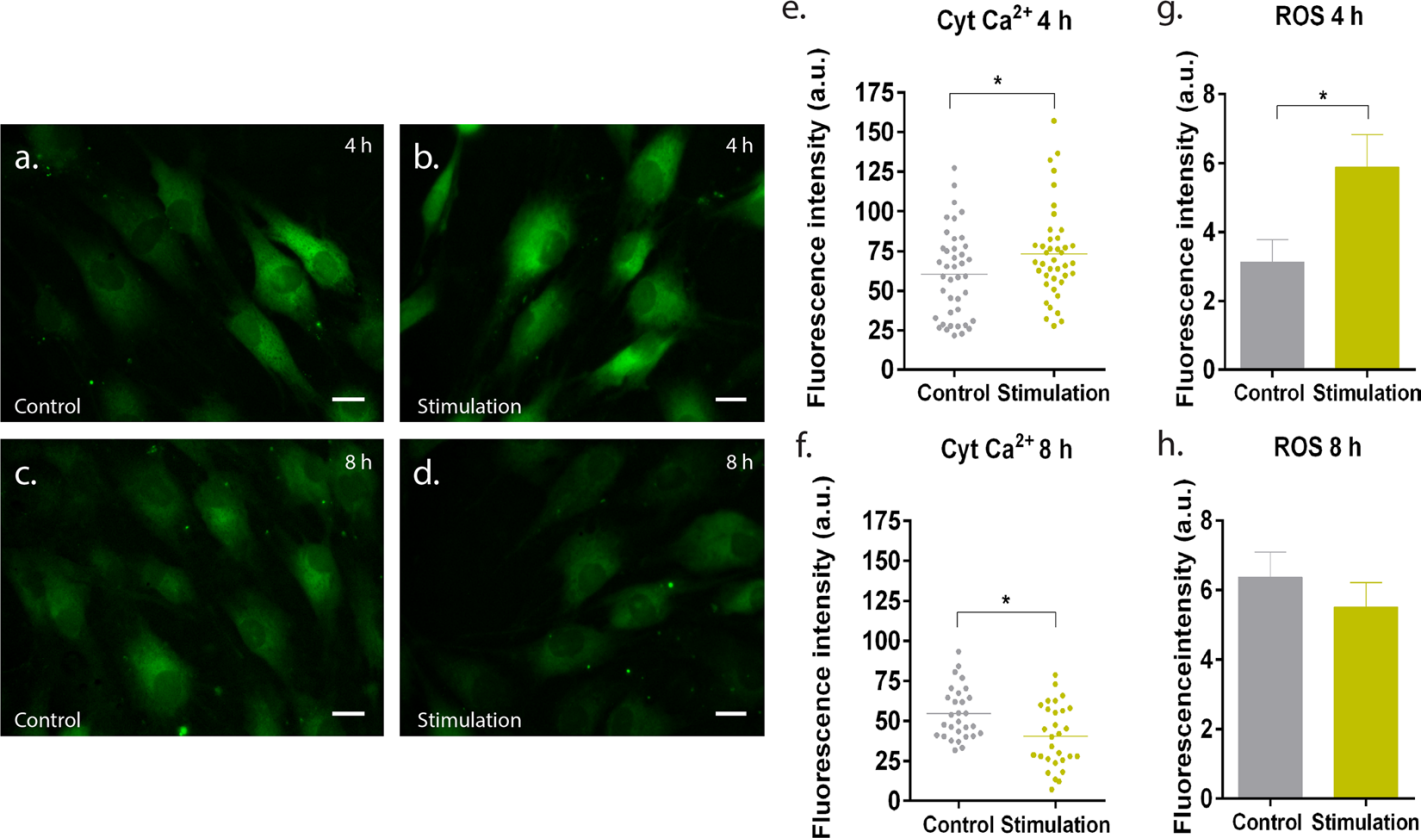
Dynamics of intracellular calcium and reactive oxygen species (ROS) in hTDCs cultured under short-term magnetic stimulation. (**a**–**d**) Detection of intracellular calcium (green) determined using the fluorescent dye Fluo-3 AM. Scale bar, 20 µm. (**e**,**f**) Quantification of fluorescence signal of intracellular calcium (cyt Ca^2+^). A minimum of 35 cells were analysed per condition and data are expressed as scattered dot plot with mean. (**g**,**h**) ROS determination according to the DCFDA assay and results are expressed as mean ± SEM (n = 9 experimental replicates from 3 biological replicates).

Intracellular Ca^2+^ was analysed as a real-time response to magnetic stimulation, being monitored using the fluorescent dye Fluo-3 AM (Fig. 1a–d). Main differences were then determined by image analysis and quantification of the fluorescence signal. In particular, magnetic stimulation during 4 h resulted in a significant increase (*p* < 0.05) in intracellular Ca^2+^ (Fig. 1e), while an 8 h-period of stimulation led to a significant decrease (*p* < 0.05) of intracellular Ca^2+^ in comparison to control cells (Fig. 1f). Accordingly, detection of reactive oxygen species (ROS) showed a similar trend (Fig. 1g,h), with a significantly higher release (*p* < 0.05) being detected in hTDCs exposed for 4 h, in comparison to control cells (Fig. 1g).

### Exposure to low-frequency static magnetic field did not affect the expression of proteins involved in mechanotransduction nor cell migration

**Figure 2:**
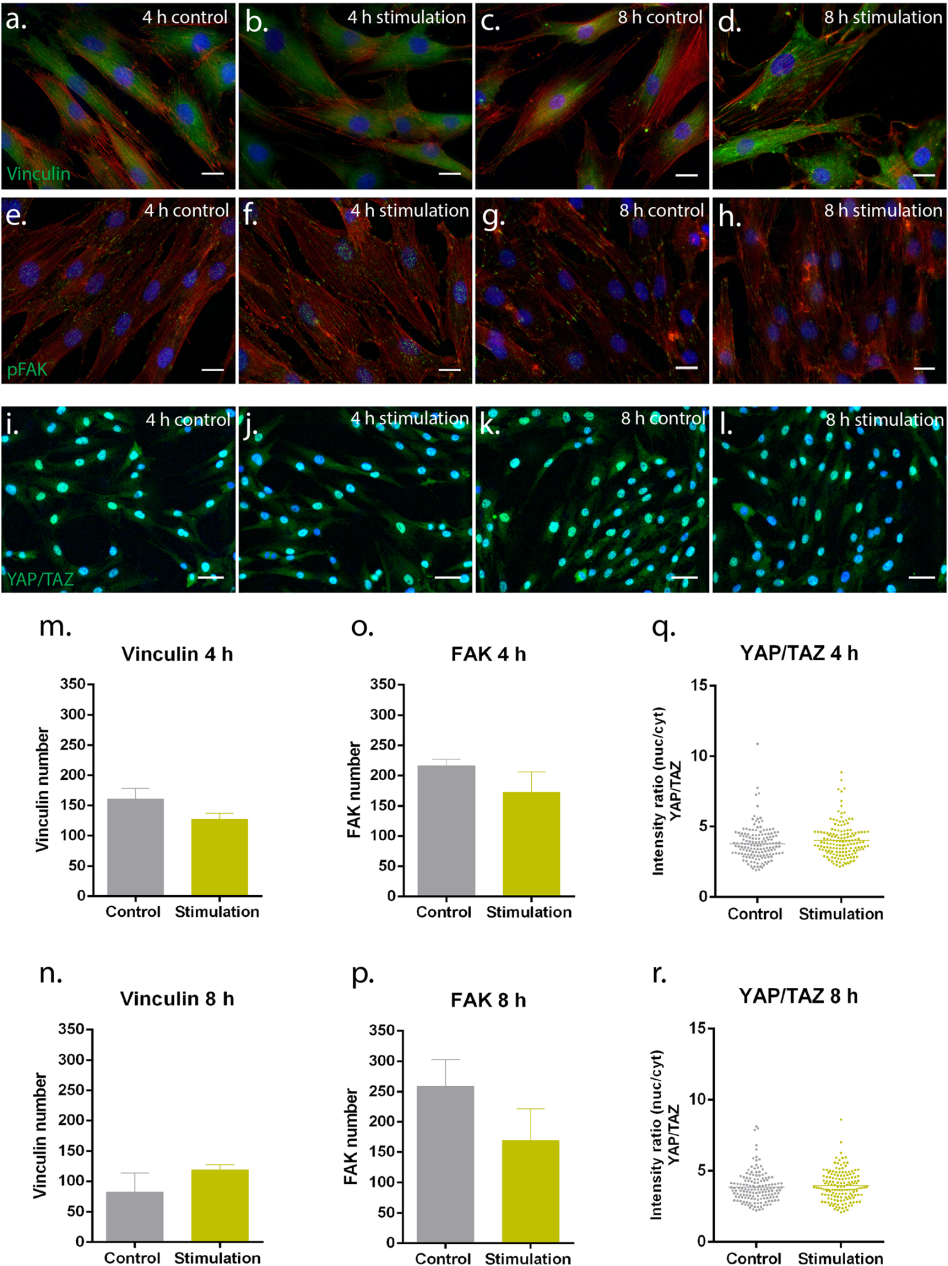
Expression of mechanosensing proteins in hTDCs cultured under short-term magnetic stimulation. (**a**–**d**) Fluorescence microscope images of immunostained vinculin (green). (**e**–**h**) Fluorescence microscope images of immunostained pFAK (green). (**a**–**h**) F-actin and nuclei were counterstained with phalloidin (red) and DAPI (blue), respectively. Scale bars, 20 μm. (**i**–**l**) Fluorescence microscope images of immunostained YAP/TAZ (green). Nuclei were counterstained with DAPI (blue). Scale bars, 50 μm. **(m–n)** Estimation of vinculin number. (**o**,**p**) Estimation of FAK number. (**m**–**p**) A minimum of 35 cells/condition were analysed and results are expressed as mean ± SEM. (**q**,**r**) Quantification of intensity ratio between nuclear and cytoplasmic expression (nuc/cyt) of YAP/TAZ. A minimum of 150 cells/condition were analysed and results are expressed as scattered dot plot with mean.

**Figure 3:**
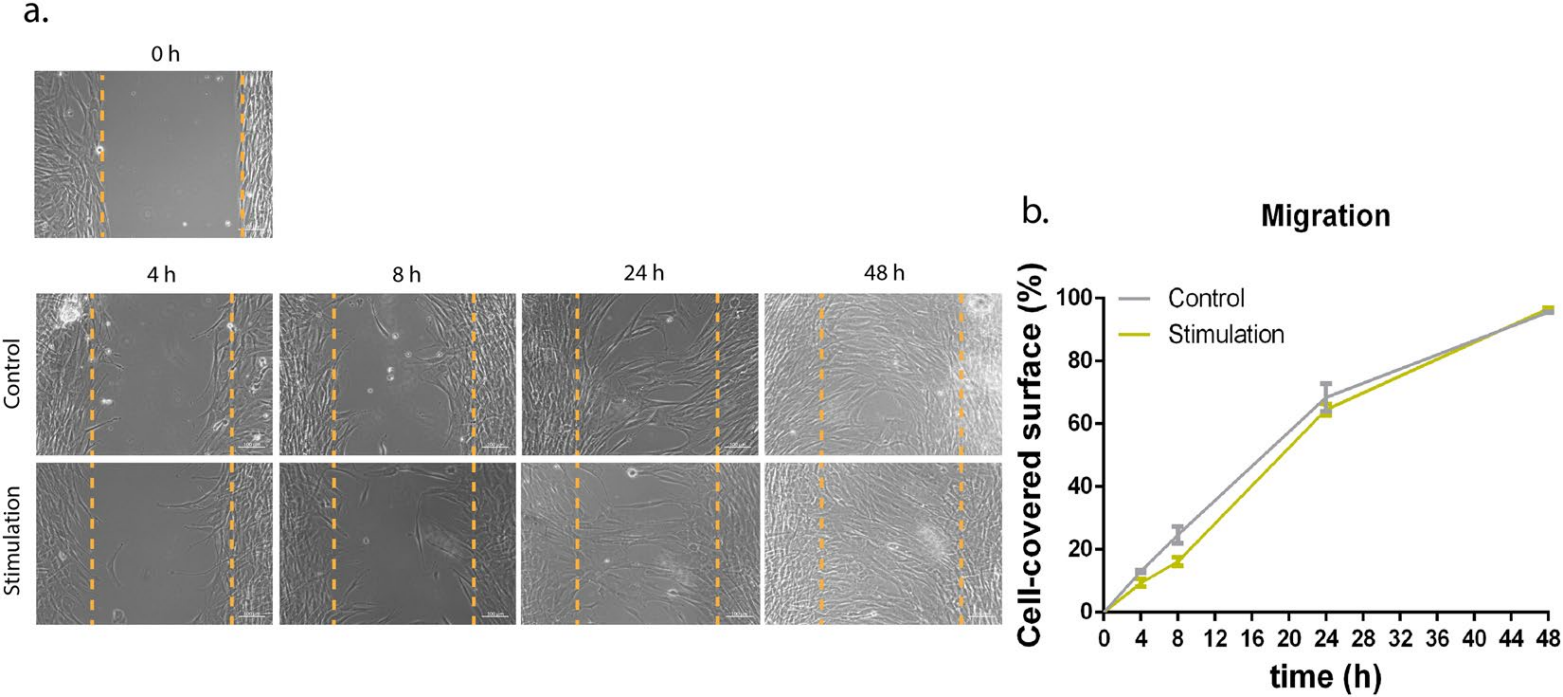
Wound closure assay under short-term magnetic stimulation. (**a**) Phase contrast photographs of the cultures taken at 0 h (immediately after scratching) and at the indicated time intervals show the wound closure by control hTDCs and hTDCs exposed to the magnetic field. (**b**) Estimation of cell coverage area.

Herein, expression of vinculin (Fig. 2a–d) and focal adhesion kinase (pFAK, Fig. 2e–h), proteins of the focal adhesion complex, and the transcriptional regulators YAP/TAZ (Fig. 2i–l) were investigated as mechanosensing proteins putatively involved in the interpretation of the magnetic signals. Protein expression evaluated by image analysis showed that the expression of vinculin (Fig. 2m,n), pFAK (Fig. 2o,p), and YAP/TAZ (Fig. 2q,r) remained similar between magnetic stimulatory conditions both at 4 h and 8 h time points. Additionally, the migratory behaviour of hTDCs up to 48 h was not influenced by exposure to a low-frequency magnetic field, as compared with control (Fig. 3a). After an initial scratching at 0 h (cell confluence), exposed cells migrate at the same rate as control cells (Fig. 3b).

### Short- and long-term magnetic stimulation altered the expression profile of tendon-associated genes

**Figure 4:**
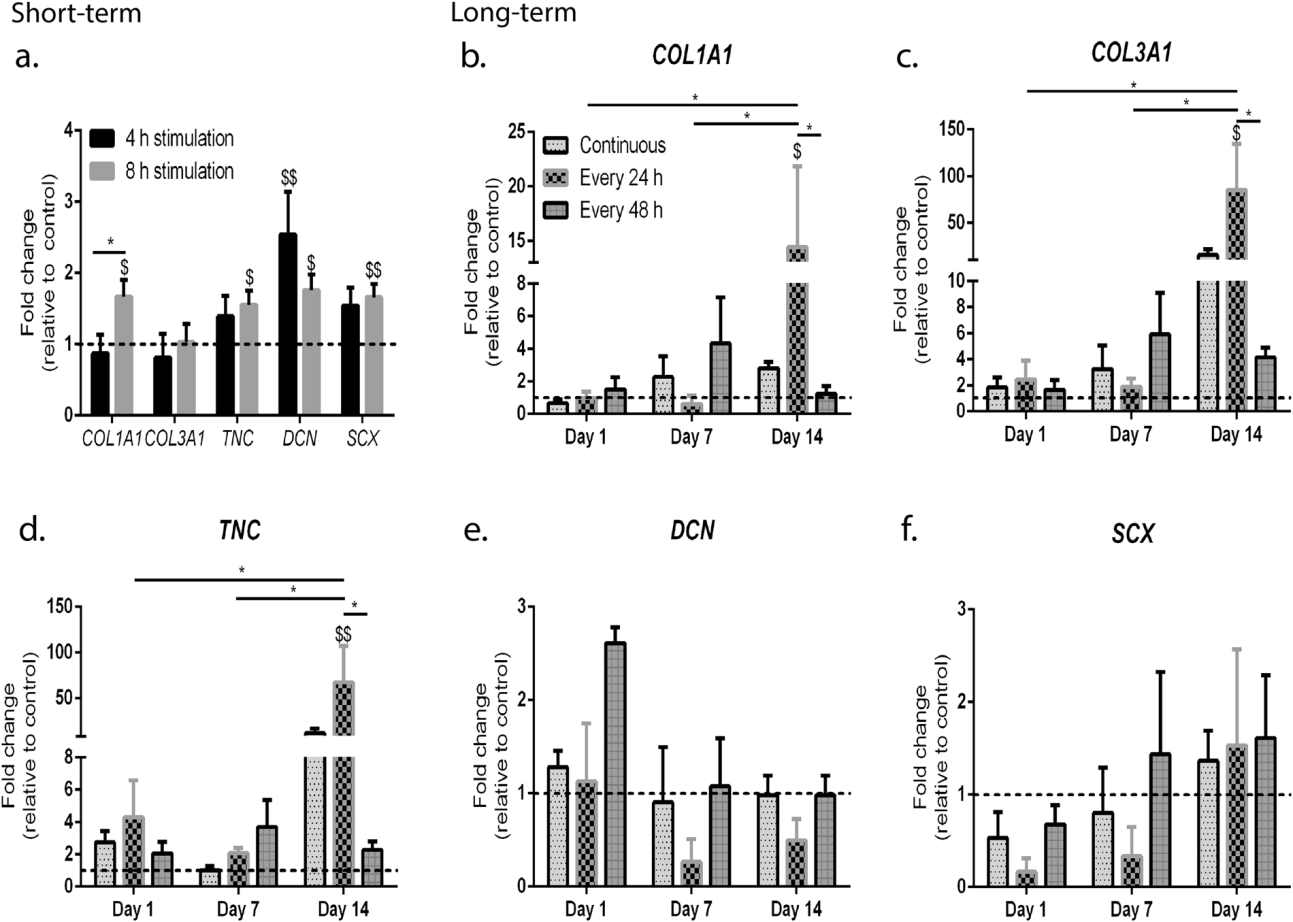
Expression of tendon-related genes in hTDCs cultured under magnetic stimulation. (**a**) Expression of *COL1A1*, *COL3A1*, *TNC*, *DCN* and *SCX* in hTDCs exposed for 4 h and 8 h to magnetic stimulation (short-term). (**b**–**f**) Expression of (**b**) *COL1A1*, (**c**) *COL3A1*, (**d**) *TNC*, (**e**) *DCN* and (**f**) *SCX* after 1, 7 and 14 days using different regimens of magnetic stimulation (long-term). Expression of target genes was normalised to *GAPDH* housekeeping gene and control cells (non-stimulated) were used as calibrator samples. Data are presented as mean ± SEM (n = 15 experimental replicates from 3 biological replicates). Statistically significant differences are shown as *p < 0.05; $ represents statistical difference in comparison to calibrator sample (^$^p < 0.05; ^$$^p < 0.005).

In order to assess the impact of the magnetic field in modulating the expression of tendon-associated genes, we studied the expression of scleraxis (*SCX*), collagen type I (*COL1A1*), collagen type III (*COL3A1*), tenascin C (*TNC*) and decorin (*DCN*) by RT-PCR following magnetic stimulation in both short- and long-term experiments (Fig. 4). Short-term exposure induced changes in the phenotype profile in a time-dependent manner, given that differences were mainly observed on hTDCs cultured for an 8 h period of magnetic stimulation (Fig. 4a). Indeed, all studied genes, with the exception of *COL3A1*, were expressed at significantly higher levels in 8 h period stimulated hTDCs, in comparison to control cells (p < 0.05 for *COL1A1*, *TNC* and *DCN*, and p < 0.005 for *SCX*, Fig. 4a). For 4 h-exposed hTDCs, only *DCN* was increased (p < 0.005 Fig. 4a). Overall, gene upregulation determined for short-term conditions was below a 3-fold change in all cases. For long-term experiments, gene expression profile seemed to be dependent on the regimen of exposure (Fig. 4b–f). Although the regulation of *DCN* (Fig. 4e) and *SCX* (Fig. 4f) was unchanged upon exposure to all conditions, the transcript levels of *COL1A1* (Fig. 4b), *COL3A1* (Fig. 4c) and *TNC* (Fig. 4d) were altered. Although no differences were found in gene regulation between days 1 and 7 of culture, the expression of *COL1A1*, *COL3A1* and *TNC* was increased from day 7 to day 14 (p < 0.05), being only significantly upregulated after 14 days in cells magnetically stimulated every 24 h (*COL1A1*, p < 0.05, Fig. 4b; *COL3A1*, p < 0.05, Fig. 4c; and *TNC*, p < 0.005, Fig. 4d) in comparison to control cells. Finally, differences between conditions were only found after 14 days of magnetic stimulation. In fact, *COL1A1*, *COL3A1* and *TNC* were upregulated in cells stimulated every 24 h, when comparing with cells stimulated every 48 h (p < 0.05).

### Short- and long-term magnetic stimulation changed the production of tendon-associated ECM proteins

**Figure 5:**
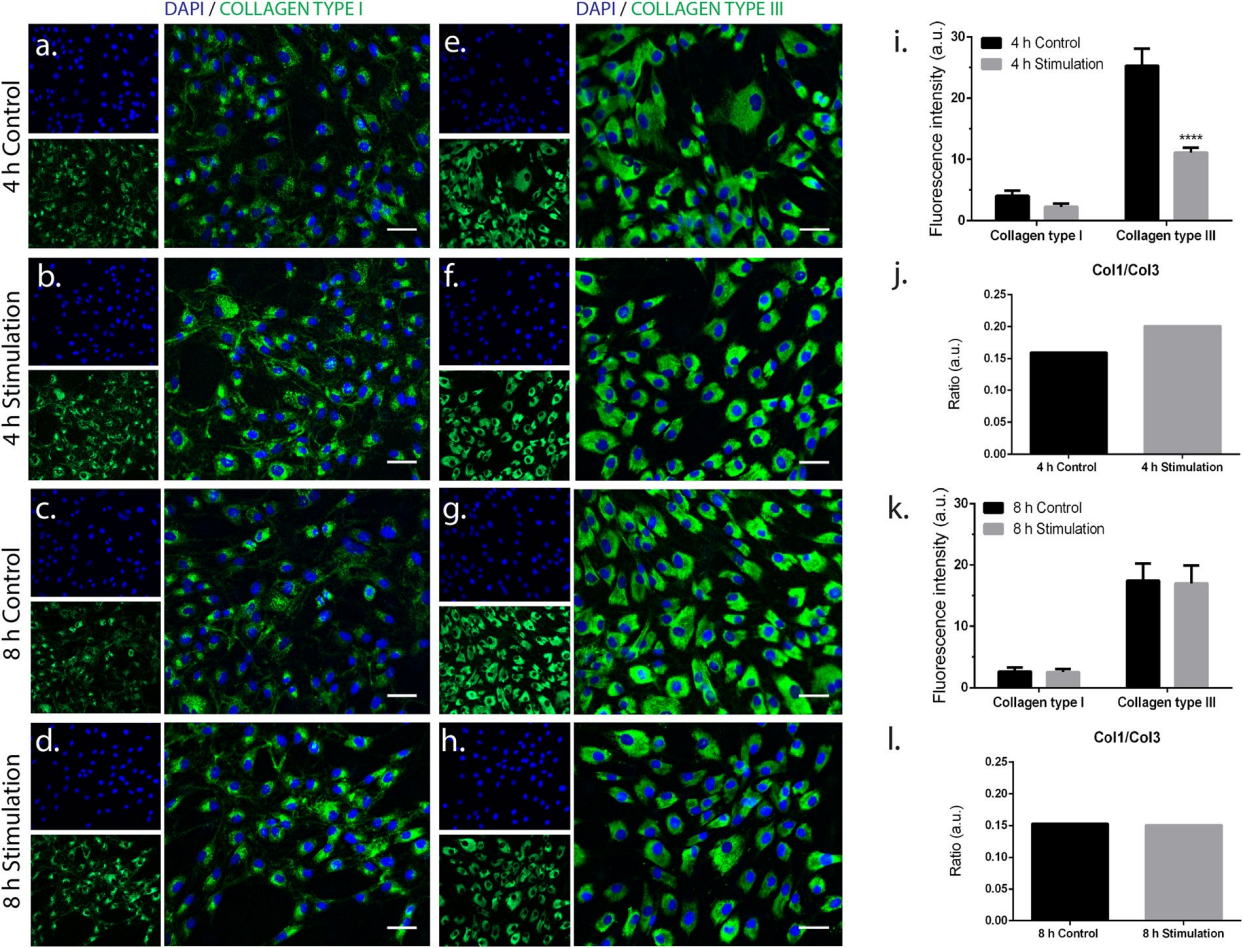
Collagen expression under short-term magnetic stimulation. (**a**–**d**) Fluorescence microscope images of immunostained collagen type I (green). (**e**–**h**) Fluorescence microscope images of immunostained collagen type III (green). Nuclei were counterstained with DAPI (blue). Scale bars, 50 μm. (**i**) Quantification of fluorescence signal of collagen types I and III and (**j**) estimation of the fluorescence ratio between collagen types I and III (ratio Col1/Col3) after 4 h of magnetic stimulation. (**k**) Quantification of fluorescence signal of collagen types I and III and (**l**) estimation of the fluorescence ratio between collagen types I and III (ratio Col1/Col3) after 8 h of magnetic stimulation. Data are expressed as mean ± SEM (n = 10 images/protein).

**Figure 6:**
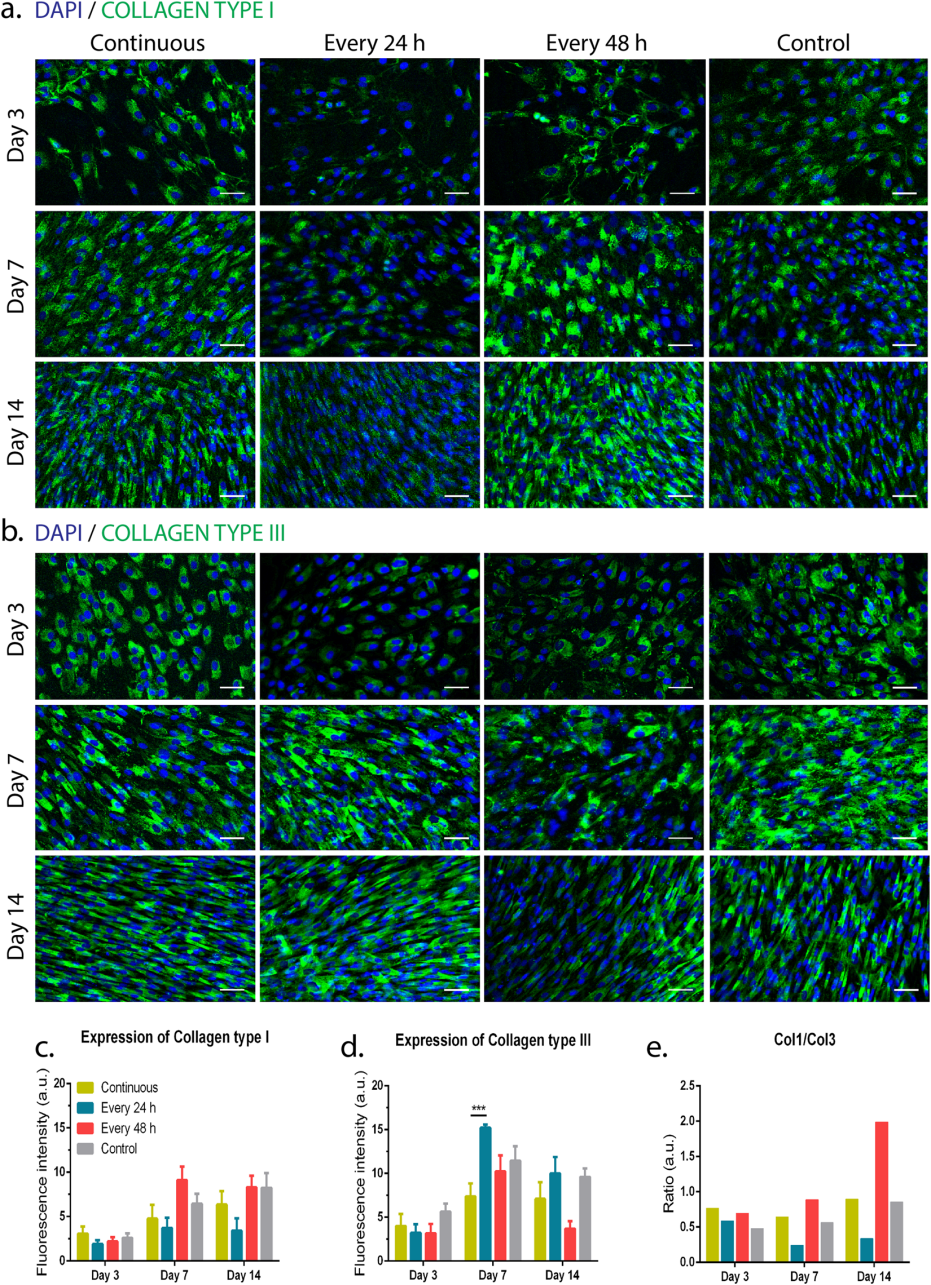
Collagen expression and deposition under long-term magnetic stimulation. (**a**) Fluorescence microscope images of immunostained collagen type I (green). (**b**) Fluorescence microscope images of immunostained collagen type III (green). (**a**,**b**) Nuclei were counterstained with DAPI (blue). Scale bars, 50 μm. Quantification of fluorescence signal of (**c**) collagen type I and (**d**) collagen type III and (**e**) estimation of the fluorescence ratio between collagen types I and III (ratio Col1/Col3) up to 14 days using different regimens of magnetic stimulation. Data are expressed as mean ± SEM (n = 10 images/protein). Statistically significant differences are shown as ***p < 0.001.

To evaluate the effect of low-frequency static magnetic field stimulation in ECM production by hTDCs, immunostainings against collagen types I and III, decorin and tenascin C were performed in both short- (Fig. 5, Supplementary Information Fig. S1) and long-term (Fig. 6, Supplementary Information Fig. S2) experiments. Initial (short-term) protein expression of collagen type I was detected at similar levels between magnetic stimulation conditions and controls both at 4 h (Fig. 5a,b,i) and 8 h (Fig. 5c,d,k). Nonetheless, the expression of collagen type III was significantly decreased in cells cultured for 4 h under magnetic stimulation in comparison to control (Fig. 5i), while no differences were detected for an 8 h-period of culture (Fig. 5k). From the expression of these two ECM proteins, the ratio between collagen type I and III in both conditions at 4 h (Fig. 5j) and 8 h (Fig. 5l) was not influenced and values range between 0.15 and 0.20.

Nevertheless, decorin and tenascin C were also expressed in all conditions likely in a similar manner (Supplementary Information Fig. S1). Synthesis and deposition of tendon-related ECM components followed an identical pattern in long-term experiments (Fig. 6). Although collagen types I (Fig. 6a) and III (Fig. 6b), as well as decorin and tenascin C (Supplementary Information Fig. S2) were expressed in all conditions, magnetic stimulation seemed to influence the type of ECM proteins being mainly produced by cultured hTDCs. Through a quantitative analysis from immunostainings, cells were found to express collagen types I (Fig. 6c) and III (Fig. 6d) at similar extents, as no significant differences were observed between magnetic stimulatory conditions and controls at day 3, 7 and 14 of culture. Moreover, a significant increase (p < 0.001) in the expression of collagen type III was detected at day 7 between cells cultured under continuous and every 24 h stimulation (Fig. 6d). Hence, cells magnetically exposed every 24 h exhibited the lowest value of col1/col3 ratio over time (Fig. 6e). In opposition, stimulating hTDCs every 48 h favoured the deposition of collagen type I over collagen type III, particularly at day 14 which resulted in the highest col1/col3 ratio (Fig. 6e). Strikingly, collagen deposition in cells continuously exposed to magnetic field was comparable to that observed for control (Fig. 6e).

### Continuous stimulation increased the secretion of the anti-inflammatory cytokine IL-10 while decreasing the release of pro-inflammatory IL-1β

**Figure 7:**
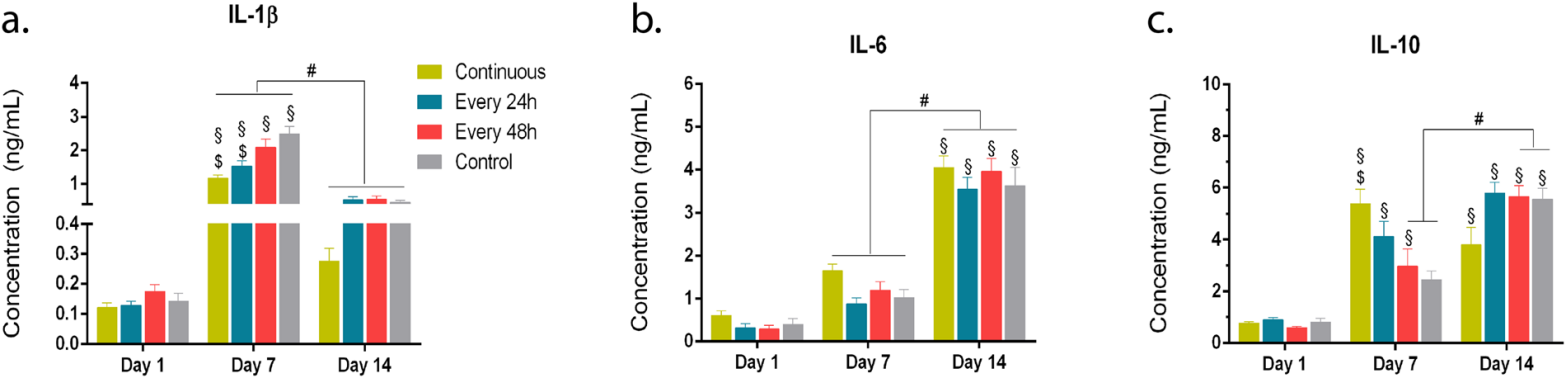
Profile of immunomodulatory cytokines secreted by hTDCs under magnetic stimulation. Quantification of (**a**) IL-1β, (**b**) IL-6 and (**c**) IL-10 present in culture media after 1, 7 and 14 days. Data are expressed as mean ± SEM (n = 5 experimental replicates from 3 biological replicates). Statistically significant differences are shown as: § symbol between Day 7/Day 14 and Day 1, # symbol between Day 14 and Day 7, and $ symbol between regimen of magnetic stimulation and control, *p* < 0.001.

To test whether the magnetic field modulated the release of pro- and anti-inflammatory cytokines, IL-1β, IL-6, and IL-10 were quantified in culture media over a period of 14 days (Fig. 7a–c). The release profile of ILs was differentially affected over time in culture under different regimens of exposure to magnetic field and controls. Moreover, no significant differences were observed among the regimens of magnetic stimulation. At day 1, no differences were found for all cytokines studied or between conditions. The release of IL-1β significantly increased from day 1 to day 7 in all conditions (p < 0.0001, 17.4-fold in control; 12-fold in every 48 h; 11.9-fold in every 24 h; 9.6-fold in continuous, Fig. 7a). At day 7, significantly lower values were detected after culturing hTDCs under continuous magnetic stimulation (1.16 ng/mL, p < 0.0001 for continuous stimulation) in comparison to control cells (2.50 ng/mL). Nonetheless, the release of IL-1β decreased significantly from day 7 to day 14 in all conditions (p < 0.0001, 5.30-fold in control; 4.20-fold in continuous; 3.8-fold in every 48 h; 2.9-fold in every 24 h), although to levels higher than those found at day 1. Overall, cells continuously stimulated exhibited the lowest IL-1β concentration over time in culture. Regarding the release profile of IL-6, the levels significantly increased from day 7 to day 14 (p < 0.0001, 4.0-fold in every 24 h, 3.54-fold in control, 3.31-fold in every 48 h, and 2.45-fold in continuous, Fig. 7b) and no differences were observed between conditions. Moreover, similarly to what was observed for IL-1β, the release of IL-10 changed under magnetic stimulation (Fig. 7c). Indeed, for all conditions, except for continuous exposure, IL-10 secretion increased over time. In particular, the IL-10 concentration was significantly higher at day 7 under magnetic stimulatory conditions in comparison to day 1 (p < 0.0001, 6.9-fold in continuous; p < 0.0001: 4.6-fold in every 24 h; p < 0.005: 5-fold in every 48 h, Fig. 7c). Strikingly, continuous stimulation induced the highest release of IL-10 (p < 0.005, 2.20-fold in comparison to control) at day 7. Between day 7 and day 14, the release of IL-10 enhanced substantially in magnetically stimulated cells every 48 h and control cells (p < 0.0001, 2.30-fold in control, 1.92-fold in every 48 h, Fig. 7c). In comparison with all stimulatory conditions and control, continuously exposed cells showed reduced expression at day 14, however, no significant difference was observed.

## Discussion

Mechanical forces are known to play a role in biological events, coordinating tissue structure and function at cellular, molecular and genetic levels. Magnetotherapy, in particular, has been receiving increased attention as an attractive strategy for the modulation of cell physiology, either through the use of magnetically actuated biomaterials^5, 18^ or by direct application of a magnetic field at the site of injury in several target tissues of the musculoskeletal system, including tendons^12–14, 19–22^. Nonetheless, there is still poor knowledge regarding the effects of magnetic field application at the cellular level and on the molecular machinery of tendon cells. Thus, in the present work, we aimed at studying the behaviour of human tendon-derived cells cultured in the presence of a low-frequency static magnetic field to gain novel insights into its effects on tenogenesis. Previous results have been described in the literature for tendon cells exposed to distinct magnetic settings, including frequencies of 75 Hz (4 h, 8 h exposure)^23, 24^ and combined frequencies of 33 Hz (10 min)/7.8 Hz (20 min)^19^, suggesting that the application of an external magnetic field does not compromise the overall viability of tendon cells. Thus, we investigated two different approaches by exposing hTDCs during short- and long-term periods of culture.

Herein, we investigated whether magnetic field stimulation, acting as a mechanical signal, affected the dynamics of molecular effectors involved in mechanotransduction and modulated the expression of mechanosensing proteins, namely vinculin and FAK, and transducers YAP/TAZ. As the time of exposure progresses, second messengers, as intracellular signalling molecules released by cells, are involved in fundamental physiological processes. Calcium is an important intracellular messenger involved in numerous cellular events, having a transient response when cells are mechanically stimulated^25^. Herein, intracellular Ca^2+^ was investigated as one of the primary molecular messengers activated in mechanotransduction events. Ca^2+^ ions enter the cell mostly through transmembrane proteins, called Ca^2+^ channels, which can either be voltage-dependent or receptor-operated^26^. In particular, L-type voltage-gated Ca^2+^ channels, which have been identified in tendon cells, are responsible for enhancing the intracellular concentration of Ca^2+^ in a time- and magnitude-dependent manner upon mechanical stimulation^27^. For instance, 12% cyclic stretching for long periods (8 h and 12 h) enhanced the intracellular calcium levels in human tendon-derived cells compared with 4 h period, as well as in comparison to 4% and 8% stretching, and unexposed cells^27^. In addition, mechanical indentation (1 Hz) for 8 h daily up to 3 days in avian tendon cells also increased the concentration of intracellular Ca^2+^ from 100 nM (basal levels) to approximately 1500 nM^28^. In this study, we observed that the dynamics of intracellular Ca^2+^ slightly changed with different times of exposure, given that Ca^2+^ levels significantly increased (1.3-fold, *p* < 0.05) after 4 h of stimulation while decreasing (1.4-fold, *p* < 0.05) upon an 8 h exposure in comparison to controls. These results suggest that different types of mechanical loading/stimulation may affect cells differently. L-type voltage-gated channels are known to be responsible to increase the intracellular concentration of calcium upon magnetic and electromagnetic stimulation^26^; however such concentration is likely to increase when cells are mechanically stimulated via activation of additional channels, such as stretch activated ion channels as a consequence of cell membrane deformation^22^. For example, a combinatory mechanical stimuli composed of cyclic tensile strain (4%) and shear stress (1666 µm/s) showed to enhance intracellular concentration in comparison with cyclic strain or shear stress alone^27^. In light with these findings, further investigation is required to understand the underlying molecular mechanism involved in calcium signalling in hTDCs exposed to magnetic field but also a detailed screening about calcium channels activated (stretch-activated *versus* voltage-gated), and to what extent calcium fluctuations impact on cytokine release and phenotypic profile.

Calcium communicates with a number of other systems and pathways^26^, including with reactive oxygen species^29^. Interactions between Ca^2+^ and ROS can be considered as bidirectional, wherein Ca^2+^ signalling is essential for the production and maintenance of homeostatic levels of ROS and, in turn, ROS can regulate intracellular Ca^2+^ signalling^30^. This mutual interaction between Ca^2+^ and ROS supports what was observed when cells were magnetically stimulated for 4 h and 8 h, as ROS production was augmented or diminished whenever there was an increase or a decrease in Ca^2+^ concentration, respectively.

To further unveil the effect of the low-frequency static magnetic field in the intracellular machinery of tendon cells, we studied the expression of two proteins important in focal adhesion complex, namely vinculin and FAK. Vinculin is a cytoplasmic actin-binding protein enriched in focal adhesions and adherent junctions involved in governing cell–matrix adhesions^31^. On the other side, FAK is a crucial signalling component that functions as a biosensor or integrator to control cell motility, which can influence the cytoskeleton, structures of cell adhesion sites and membrane protrusions to regulate cell movement^32^. Tyrosine phosphorylation of FAK, FAK Tyr-397, assists in the recruitment and binding of focal adhesion proteins by regulating protein-protein interactions^33^. Herein, no significant differences were observed in the expression of vinculin and FAK under magnetic stimulatory conditions compared with control after 4 h and 8 h of culture. Moreover, the conversion of physical stimuli into biochemical signals takes place via coordinated intracellular events, which involve multiple complexes and signalling molecules. The transcriptional regulators YAP/TAZ, core of the canonical Hippo signalling pathway, have been shown to act as transducers of physical forces^34^, for example, mechanical input involving substrate stiffness^35^ or cyclic stretching dependent signals^36^. Our analysis focused on testing whether YAP/TAZ would be involved in the conversion of magnetic waves in hTDCs stimulated with a low-frequency static magnetic bioreactor. By keeping the substrate stiffness constant (cell culture coverslips), the ratio between nuclear and cytoplasmic expression of YAP/TAZ was assessed following 4 h and 8 h of stimulation and in controls. Our result showed that YAP/TAZ expression remained unaltered for every tested condition and time point. Mechanistically, studies reported that uniaxial cyclic stretching (1 Hz, 20% strain), as physical stimuli, applied for a 6 h period was able to increase the nuclear localisation of YAP (80%) over cytoplasmic localisation (30%) in human mammary epithelial cell line MCF10A^36^ or periodic cyclic stretching (0.1 Hz, 5% strain) for the same period but distinctive substrates triggered the translocation of YAP from cytoplasm towards nuclei in primary mouse embryonic fibroblasts^37^. In the present study, low-frequency magnetic field (2 Hz) and the static mode of presenting the signal to hTDCs may not be sufficient to increase intracellular tensions as felt by cells during stretching, therefore appointed as one of the reasons for identical expression levels of YAP/TAZ in magnetically stimulating conditions and controls^38^, as well as similar expression patterns of integrin-dependent focal adhesions mechanosensing proteins, vinculin and FAK.

Next, we sought to understand if there was any correlation between the expression profiles of vinculin and FAK in the migration ability of tendon cells. As part of the mechanosensing complex, FAK Tyr-397 is required for some of the functions of FAK in cell migration^39^. In our study, the migratory behaviour of tendon cells was not significantly affected by exposure to magnetic field, further confirming the results regarding the expression of the mechanosensing proteins studied herein.

While the expression of mechanosensing proteins and the migratory profile was similar between stimulatory conditions and controls, we showed here that low-frequency static magnetic field influenced the phenotypic profile of hTDCs. Initially, 8 h period in short-term exposure significantly increased the expression of tendon-related genes *SCX* (p < 0.01), *COL1A1* (p < 0.05), *DCN* (p < 0.05) and *TNC* (p < 0.05) in comparison to control. Additionally, to gain further insights into the influence of magnetic field on the phenotype of hTDCs, the expression of tendon-related genes was evaluated in long-term studies (up to 14 days). Exposing hTDCs to an 8 h period every 24 h significantly upregulated (p < 0.05) the expression of *COL1A1*, *COL3A1* and *TNC* after 14 days in culture. Interestingly, such an increase was not observed in any other regimen of exposure (continuous and every 48 h) nor controls overtime, suggesting that, for the same magnetic settings used, the regimen of exposure is of importance when modulating the phenotypic profile of hTDCs. Other studies showed that exposing tendon-derived cells to 75 Hz of pulsed electromagnetic field for 2 days did not trigger any changes in the expression of *SCX*, *COL1A1* and *COL3A1* ^23, 24^ while the expression of *DCN* and *TNC* was not studied. Therefore, the regimen of exposure, as well as magnetic settings used including frequency, type of exposure (static *versus* pulsed), type of stimulatory field (magnetic *versus* electromagnetic), magnetic flow and magnetic gradient may affect cell physiology.

Furthermore, variations in the deposition levels of collagen type I and type III overtime, when cells were stimulated every 48 h using the same window (8 h) of magnetic stimulation, resulted in an increase of the ratio between collagen types I and III (col1/col3 ratio), particularly from day 7 to day 14, consequently suggesting that this magnetic setting is likely to favour a pro-healing state in tendon cells^40^.

Despite not being directly comparable, the magnetic settings that we used in the present work could be of beneficial interest in driving stem cell commitment into the tenogenic lineage as observed in mechanical loaded systems^1^. For example, culturing tendon stem and progenitor cells on poly(L-lactide-co-ε-caprolactone)/collagen fibrous scaffold and cyclic stretched at a 0.5 Hz frequency with an amplitude of 4% enhanced gene expression of *Col1a1*, *Tnc*, *Scx*, *Tnmd* ^1^.

Furthermore, we found that the regimen of exposure also played a role in the release of pro- and anti-inflammatory cytokines. Indeed, a long-term continuous magnetic stimulation significantly decreased the release of IL-1β while increasing the release of IL-10, particularly following 7 days of magnetic stimulation. Upon injury, tendons follow several phases during healing involving multiple biologically active molecules, including inflammatory factors^41^. Particularly, culturing tendon cells for 2 days using electromagnetic stimulation has been shown to increase the release of the anti-inflammatory cytokine IL-10 while decreasing the release of IL-1β^23, 24^. Results obtained in the present study also showed a down-regulation of IL-1β and up-regulation of IL-10 after 7 days in culture, suggesting that a prolonged continuous exposure is likely to favour an anti-inflammatory state in tendon cells. Indeed, an upregulation of IL-10 has been correlated with an enhanced healing in murine models after patellar tendon injury^42^. Collectively, these results showed that different regimens of magnetic exposure have distinct effects on tendon cell physiology as an exposure to 8 h-period of magnetic stimulation every 24 h was beneficial to preserve tenogenesis. On the other side, an exposure every 48 h favoured collagen deposition (increased col1/col3 ratio) while continuous stimulation is more likely to maintain the balance between IL-1β and IL-10.

As a contact-free technology and simultaneously acting both as a mechanical stimulation and modulation of the inflammatory response, of particular importance for tendon regeneration, we foresee that magnetic field can represent an interesting candidate to remotely guide tissue regeneration.

## Conclusion

The physiology of tendon cells is intrinsically influenced by exposure to mechanical forces. In tendon, magnetic field therapy has emerged as an alternative contact-free technology to enhance tissue healing through modulation of inflammatory response. However, the behaviour of tendon cells under such magnetic fields is still relatively unexplored. In the present work, we have succeeded to unveil that low-frequency static magnetic field modulates the concentration of intracellular calcium, as well as the release of ROS in a time-dependent manner. Moreover, a short-term exposure of 8 h upregulated tendon-related genes, *SCX*, *COL1A1*, *TNC* and *DCN*, while in long-term stimulation, an 8 h magnetic window applied every 24 h significantly increased the expression of *COL1A1*, *COL3A1* and *TNC*. Additionally, the balance between the release of IL-1β and IL-10 was attained with continuous stimulation. Overall, magnetic actuation can potentially be used to support the application of magnetic biomaterials in modulating tendon regeneration.

## Methods

### Isolation and expansion of human tendon-derived cells (hTDCs)

Tendon tissue was collected from patients undergoing elective orthopaedic surgeries at Hospital da Prelada (Porto, Portugal) under informed consent and according to protocols approved by the Ethical Committee of Hospital da Prelada. Herein, human tendon-derived cells (hTDCs) were isolated as previously described^43^ using three healthy tendon samples (autograft) collected from the knee (Sartorius) of male patients with ages in the range of 25–30 years. Shortly, tissue samples were rinsed in phosphate buffered saline (PBS) solution containing 10% (v/v) of antibiotic/antimitotic (AB/AM, Life Technologies) and tendon tissue was carefully isolated. Tendon was minced and then digested in 0.1% (w/v) type I collagenase solution (collagenase from *Clostridium histolyticum* release of physiologically active rat epididymal adipocyte, Sigma-Aldrich, 125 collagen digestion unit (CDU)/mg) at 37 °C for at least 1 h in an orbital shaker at 200 rpm. Following filtration (100 μm filter) and double centrifugation at 4 °C for 5 min each cycle at 1250 rpm, the supernatant was discarded and the cell pellet suspended in Minimum Essential Medium alpha (α-MEM, Invitrogen) supplemented with 10% (v/v) fetal bovine serum (FBS, Alfagene) and 1% (v/v) AB/AM. Cells were incubated in standard humidified atmosphere of 5% CO_2_ at 37 °C. Cells were used between passages 3 and 5 in all experiments.

### Cell culture and magnetic stimulation

#### Short-term studies

Human TDCs were cultured at initial densities of 25,000 cells/cm^2^ (for all assays) in supplemented α-MEM in standard atmosphere for 24 h. Cells were then exposed for 4 h and 8 h to a magnetic field using a magnetic force system (Nanotherics, 2 Hz oscillatory frequency, 350 mT magnetic field force, 0.2 mm displacement).

#### Long-term studies

Human TDCs were cultured at initial densities of 5,000 (for immunostaining) and 25,000 cells/cm^2^ (for all the other assays) in supplemented α-MEM up to 14 days. Cell culture followed the same conditions aforementioned. Different groups were considered for magnetic stimulation: (i) continuous stimulation, (ii) every 24 h, (iii) every 48 h and (iv) non-stimulated cells (Control). An 8-hour period of stimulation was chosen when hTDCs were either exposed every 24 h or 48 h. Fresh culture medium was added every two days.

### Cell migration profile

Cells were cultured at a density of 114,000 cells/cm^2^ *per* well (2 well silicone insert, Ibidi®, gap size of 500 µm) in supplemented α-MEM in standard atmosphere for 24 h. After 24 h of culture, cells achieved confluency, the silicone insert was removed and cells were observed and photographed using a light microscope (Zeiss). Thereafter, they were cultured under low-frequency magnetic stimulation and standard conditions. Cell migration was then observed using light microscope at 4 h, 8 h, 24 h and 48 h. Images of migration profile were analysed by ImageJ software. Cell migration was expressed as cell-covered surface: (original scratch area - new scratch area)/original scratch area × 100%.

### Intracellular calcium

Cells were initially washed with Hank’s Buffer with 20 mM HEPES solution (AAT Bioquest) and incubated with Hank’s buffer containing 5 μM of Fluo-3/AM (39294, Sigma-Aldrich) and 1 mg/mL of Pluronic® F-127 (Sigma-Aldrich) for 1 h in standard atmosphere in the dark, followed by 30 min with dye free Hank’s buffer^27, 44^. Stained cells were analysed by fluorescence microscopy.

### Reactive oxygen species (ROS)

ROS was quantified via DCFDA Cellular ROS Detection Assay (ab113851, Abcam). Cells were stained with 20 μM of DCFDA compound and incubated in standard humidified atmosphere for 45 min. The fluorescence signal of the supernatant in the presence of respective compound was measured using a microplate reader (Synergy^TM^ HT, BIO-TEK Instruments, Excitation 485 nm and Emission 530).

### Quantitative reverse transcription PCR (RT-PCR) analysis

Total mRNA was isolated from cells utilising TRI Reagent® RNA Isolation Reagent (Sigma-Aldrich) and analysed using NanoDrop 1000 Spectrophotometer (ThermoFisher Scientific). Then, mRNA (500 pg) was utilised to transcribe the information into complementary DNA (cDNA) using qScript^TM^ cDNA Synthesis Kit (Quanta BioSciences). The obtained cDNA was bound to fluorescent dye SYBR Green I from PCR master kit (Quanta BioSciences) and the analysis carried out on RT-PCR Mastercycler (Realplex, Eppendorf). Gene expression profile was normalised to the expression of the reference housekeeping gene GAPDH according to Livak’s method^45^. Results are represented as fold change. Primer sequences are listed in Supplementary Table S1 and were designed using Primer-BLAST tool. The oligonucleotides were synthesised by Eurofins Genomics (UK).

### Immunocytochemical analysis

Immunocytochemical analysis was performed following 4 h and 8 h (short-term studies) and after 3, 7 and 14 days (long-term studies). Cells were fixed with 10% (v/v) neutral buffered formalin (Richard-Allan Scientific^TM^, ThermoFisher Scientific) for 20 min, permeabilized with 0.25% (v/v) of Triton X-100 (Sigma-Aldrich) for a period ranging from 10 to 30 min, for extracellular and intracellular protein expression, respectively. Cells were incubated with 1% (w/v) bovine serum albumin (BSA, Sigma-Aldrich) for 30 min. To evaluate the expression of intracellular proteins, cells were incubated with primary antibodies against YAP/TAZ (mouse monoclonal, 1:200, sc101199, SantaCruz Biotechnology), FAK (rabbit polyclonal, 5 μg/mL, ab39967, Abcam), and vinculin (mouse monoclonal, 1:100, V9131, Sigma-Aldrich) at 4 °C overnight. For studying extracellular proteins, cells were incubated with primary antibodies against Collagen type I (rabbit polyclonal, 1:500, ab34710, Abcam), Collagen type III (rabbit polyclonal, 1:100, ab7778, Abcam), Tenascin C (mouse monoclonal, 1:3000, ab6393, Abcam) and Decorin (mouse monoclonal, 1:100, ab54728, Abcam) for 1 h. Cells were then incubated with secondary antibodies Alexa Fluor® 488 donkey anti-rabbit IgG (H+L) (1:1000, A21206, Molecular Probes®), Alexa Fluor® 488 donkey anti-mouse IgG (H+L) (1:300 (only for YAP/TAZ) or 1:1000, A21202, Molecular Probes®). Actin filaments were stained with Phalloidin (1:200, P1951, Sigma-Aldrich) for 30 min and nuclei were counterstained with DAPI (1:1000, D9542, Sigma-Aldrich) for 5 min. All antibodies were diluted in 1% (v/v) BSA; phalloidin and DAPI were prepared in PBS. Cells were washed at least three times between incubation steps. Stained cells were analysed by fluorescence microscopy.

### Quantitative analysis of fluorescence images

All fluorescence images were obtained using a fluorescence microscope (Transmitted and Reflected Light Microscope with Apoptome 2, Zeiss Group). The fluorescence signal emitted from the expression of the intended proteins was quantified using ImageJ software. Following image acquisition (n = 10 images/protein), the green channel was background corrected and the Huang correction method for distinction between background and foreground was used^46^. Then, the emission intensity of the protein was measured^47^ according to Equation (1):

1 $${\rm{Protein}}\,{\rm{intensity}}={\rm{Protein}}\,{\rm{signal}}-({\rm{Protein}}\,{\rm{area}}\times {\rm{Background}}\,{\rm{signal}})$$

where the protein signal is the sum of the intensity of the pixels defined as foreground and the background signal corresponds to the mean signal for the selected region excluding the protein signal.

### Enzyme-linked Immunosorbent Assay (ELISA)

The release of immunomodulatory cytokines into cell medium supernatant was evaluated at day 1, 7 and 14 of culture (long-term studies) by sandwich ELISA detection method for interleukin (IL)-1β (Human IL-1β Standard ABTS ELISA Development Kit, 900-K95, PeproTech, London, UK), IL-6 (Human IL-6 Standard ABTS ELISA Development Kit, 900-K16, PeproTech, London, UK) and IL-10 (Human IL-10 Standard ABTS ELISA Development Kit, 900-K21, PeproTech, London, UK), using 2,2′-Azino-bis(3-ethylbenzothiazoline-6-sulfonic acid) (ABTS, Sigma-Aldrich, St. Louis, US) as the reaction substrate. IL detection was performed according to manufacturer’s instructions and by incubating standards/samples overnight. The optical density was monitored using a microplate reader (Synergy^TM^ HT, BIO-TEK Instruments, Absorbance 405/650 nm). For ELISA assay, fresh culture medium was added to cell culture every 48 h after day 1. At the time-points selected, culture media were collected and data was analysed for each time point individually.

### Statistical analysis

Statistical analysis was conducted using GraphPad Prism v6.0 software. Results were normalised to control and are expressed as mean ± SEM. The n-values describe the experimental replicates used for each of the analysis, fully detailed in each of the figures.

The statistical analyses were performed using Student’s t-test or one-way ANOVA test with Tukey posthoc test. Statistical significances were established for *p* values < 0.05.

### Data availability statement

All data generated or analysed during this study are included in this published article (and its Supplementary Information files).

## Electronic supplementary material

Supplementary Information

## References

[1] Xu, Y. *et al*. Cyclic Tensile Strain Induces Tenogenic Differentiation of Tendon-Derived Stem Cells in Bioreactor Culture. *Biomed Res*. *Int*. **2015** (2015). 10.1155/2015/790804 PMC4502284 26229962

[2] Schweitzer R (2001). Analysis of the tendon cell fate using Scleraxis, a specific marker for tendons and ligaments. Development.

[3] Shukunami C, Takimoto A, Oro M, Hiraki Y (2006). Scleraxis positively regulates the expression of tenomodulin, a differentiation marker of tenocytes. Dev. Biol..

[4] Zhang J, Wang JHC (2010). Mechanobiological response of tendon stem cells: Implications of tendon homeostasis and pathogenesis of tendinopathy. J. Orthop. Res..

[5] Gonçalves AI (2016). Exploring the Potential of Starch/Polycaprolactone Aligned Magnetic Responsive Scaffolds for Tendon Regeneration. Adv. Healthc. Mater..

[6] Silva, E. D., Gonçalves, A. I., Santos, L. J., Rodrigues, M. T. & Gomes, M. E. In *Smart Materials for Tissue Engineering Fundamental Principles* 491–520 (2017).

[7] Wang JH, Li B (2010). Mechanics rules cell biology. Sports Med. Arthrosc. Rehabil. Ther. Technol..

[8] Markov MS (2007). Expanding use of pulsed electromagnetic field therapies. Electromagn. Biol. Med..

[9] Jaberi FM (2011). A Moderate-Intensity Static Magnetic Field Enhances Repair of Cartilage Damage in Rabbits. Arch. Med. Res..

[10] Meng J (2013). Super-paramagnetic responsive nanofibrous scaffolds under static magnetic field enhance osteogenesis for bone repair *in vivo*. Sci. Rep..

[11] Russo A (2016). Magnetic forces and magnetized biomaterials provide dynamic flux information during bone regeneration. J. Mater. Sci. Mater. Med..

[12] Xu D, Zhang T, Qu J, Hu J, Lu H (2014). Enhanced patella-patellar tendon healing using combined magnetic fields in a rabbit model. Am. J. Sports Med..

[13] Binder, A., Parr, G. & Hazleman, B. Pulsed Electromagnetic Field Therapy of Persisted Rotator Cuff Tendinitis. *Lancet* (1984). 10.1016/s0140-6736(84)92219-0 6143039

[14] Strauch B (2006). Pulsed magnetic field therapy increases tensile strength in a rat Achilles’ tendon repair model. J. Hand Surg. Am..

[15] Santos L (2016). *In vitro* and *in vivo* assessment of Magnetically Actuated Biomaterials and Prospects in Tendon Healing. Nanomedicine (Lond)..

[16] Lima J, Gonçalves AI, Rodrigues MT, Reis RL, Gomes ME (2015). The effect of magnetic stimulation on the osteogenic and chondrogenic differentiation of human stem cells derived from the adipose tissue (hASCs). J. Magn. Magn. Mater..

[17] Silva, E. D. *et al*. Multifunctional magnetic-responsive hydrogels to engineer tendon-to-bone interface. *Nanomedicine Nanotechnology*, *Biol*. *Med*. 1–11, doi:10.1016/j.nano.2017.06.002 (2017). 10.1016/j.nano.2017.06.002 28614734

[18] Cezar CA (2016). Biologic-free mechanically induced muscle regeneration. Proc. Natl. Acad. Sci. USA.

[19] Seeliger C, Falldorf K, Sachtleben J, van Griensven M (2014). Low-frequency pulsed electromagnetic fields significantly improve time of closure and proliferation of human tendon fibroblasts. Eur J Med Res.

[20] Lee EWC, Maffulli N, Li CK, Chan KM (1997). Pulsed magnetic and electromagnetic fields in experimental Achilles tendonitis in the rat: A prospective randomized study. Arch. Phys. Med. Rehabil..

[21] Denaro V (2011). Effect of pulsed electromagnetic fields on human tenocyte cultures from supraspinatus and quadriceps tendons. Am. J. Phys. Med. Rehabil..

[22] Randelli, P. *et al*. Effects of the pulsed electromagnetic field PST ® on human tendon stem cells: a controlled laboratory study. *BMC Complement*. *Altern*. *Med*. 1–11 doi:10.1186/s12906-016-1261-3 (2016). 10.1186/s12906-016-1261-3 PMC4989537 27538432

[23] de Girolamo L (2013). Low Frequency Pulsed Electromagnetic Field Affects Proliferation, Tissue-Specific Gene Expression, and Cytokines Release of Human Tendon Cells. Cell Biochem. Biophys..

[24] de Girolamo L (2014). *In vitro* functional response of human tendon cells to different dosages of low-frequency pulsed electromagnetic field. Knee Surgery, Sport. Traumatol. Arthrosc..

[25] Wall ME, Banes AJ (2005). Early responses to mechanical load in tendon: Role for calcium signaling, gap junctions and intercellular communication. J. Musculoskelet. Neuronal Interact..

[26] Berridge MJ, Lipp P, Bootman MD (2000). The versatility and universality of calcium signalling. Nat. Rev. Mol. Cell Biol..

[27] Chen W, Deng Y, Zhang J, Tang K (2015). Uniaxial repetitive mechanical overloading induces influx of extracellular calcium and cytoskeleton disruption in human tenocytes. Cell Tissue Res..

[28] Banes, A. J. *et al*. Gap Junctions Regulate Responses of Tendon Cells *Ex Vivo* to Mechanical Loading. *Clin*. *Orthop*. *Relat*. *Res*. 356–370 (1999). 10.1097/00003086-199910001-00034 10546659

[29] Görlach A, Bertram K, Hudecova S, Krizanova O (2015). Calcium and ROS: A mutual interplay. Redox Biol..

[30] Gordeeva AV, Zvyagilskaya RA, Labas YA (2003). Review: Cross-talk between reactive oxygen species and calcium in living cells. Biokhimiya.

[31] Peng, X., Nelson, E. S., Maiers, J. L. & DeMali, K. A. *New Insights into Vinculin Function and Regulation*. *International Review of Cell and Molecular Biology* **287** (Elsevier Inc., 2011). 10.1016/B978-0-12-386043-9.00005-0 PMC4426885 21414589

[32] Mitra SK, Hanson Da, Schlaepfer DD (2005). Focal adhesion kinase: in command and control of cell motility. Nat. Rev. Mol. Cell Biol..

[33] Calalb MB, Polte TR, Hanks SK (1995). Tyrosine Phosphorylation of Focal Adhesion Kinase at Sites in the Catalytic Domain Regulates Kinase Activity: a Role for Src Family Kinases. Mol. Cell. Biol..

[34] Piccolo S, Dupont S, Cordenonsi M (2014). The Biology of YAP/TAZ: Hippo Signaling and Beyond. Physiol. Rev..

[35] Dupont S (2011). Role of YAP/TAZ in mechanotransduction. Nature.

[36] Codelia VA, Sun G, Irvine KD (2014). Regulation of YAP by mechanical strain through Jnk and Hippo signaling. Curr. Biol..

[37] Cui Y (2015). Cyclic stretching of soft substrates induces spreading and growth. Nat. Commun..

[38] Halder G, Dupont S, Piccolo S (2012). Transduction of mechanical and cytoskeletal cues by YAP and TAZ. Nat. Publ. Gr..

[39] Wang HB, Dembo M, Hanks SK, Wang Y (2001). Focal adhesion kinase is involved in mechanosensing during fibroblast migration. Proc. Natl. Acad. Sci. USA.

[40] Lui PPY, Chan LS, Lee YW, Fu SC, Chan KM (2010). Sustained expression of proteoglycans and collagen type III/type I ratio in a calcified tendinopathy model. Rheumatology.

[41] Nourissat G, Berenbaum F, Duprez D (2015). Tendon injury: from biology to tendon repair. Nat. Rev. Rheumatol..

[42] Ricchetti ET (2008). Effect of Interleukin-10 Overexpression on the Properties of Healing Tendon in a Murine Patellar Tendon Model. J. Hand Surg. Am..

[43] Costa-Almeida, R. *et al*. Microengineered Multicomponent Hydrogel Fibers: Combining Polyelectrolyte Complexation and Microfluidics. *ACS Biomater*. *Sci*. *Eng*. doi:10.1021/acsbiomaterials.6b00331 (2016). 10.1021/acsbiomaterials.6b00331 33429690

[44] Meng G (2014). Temperature-induced labelling of Fluo-3 AM selectively yields brighter nucleus in adherent cells. Biochem. Biophys. Res. Commun..

[45] Livak KJ, Schmittgen TD (2001). Analysis of relative gene expression data using real-time quantitative PCR and. Methods.

[46] Chen S, Leung H (2004). Survey over image thresholding techniques and quantitative performance evaluation. J. Electron. Imaging.

[47] Gavet O, Pines J (2010). Progressive Activation of CyclinB1-Cdk1 Coordinates Entry to Mitosis. Dev. Cell.

