# Supplementary material for: Uncovering the effect of low-frequency static magnetic field on tendon-derived cells: from mechanosensing to tenogenesis
Source: Sci Rep. 2017 Sep 8;7:10948. doi: 10.1038/s41598-017-11253-6 (PMC5591251; doi:10.1038/s41598-017-11253-6)
Supplement: Supplementary file 1 — Supplementary Information [file 41598_2017_11253_MOESM1_ESM.pdf]

Uncovering the effect of low-frequency static magnetic field on tendon-derived cells: from mechanosensing to tenogenesis

Tamagno Pesqueira [1,2#], Raquel Costa-Almeida [1,2#], Manuela E. Gomes [1,2*]

[1] 3B's Research Group – Biomaterials, Biodegradables and Biomimetics, University of Minho, Headquarters of the European Institute of Excellence on Tissue Engineering and Regenerative Medicine, Avepark – Parque de Ciência e Tecnologia, Zona Industrial da Gandra, 4805-017 Barco, Guimarães, Portugal

[2] ICVS/3B's – PT Government Associate Laboratory, Guimarães, Portugal

[#] These authors contributed equally to this work

***Corresponding author:** megomes@dep.uminho.pt

Supplementary Information

Supplementary Table S1 - Gene sequence for RT-PCR analysis

**Target gene**	**Primer Sequence**	**NCBI reference**
Glyceraldehyde-3-phosphate dehydrogenase (*GAPDH*)	F: TGTACCACCAACTGCTTAGC	NM 002046.4
	R: GGCATGGACTGTGGTCATGAG	
Scleraxis (*SCX*)	F: AGAACACCCAGCCCAAACAGAT	NM 001080514.2
	R: TCGCGGTCCTTGCTCAACTTT	
Tenascin C (*TNC*)	F: ACTGCCAAGTTCACAACAGACC	NM 002160.3
	R: CCCACAATGACTTCCTTGACTG	
Decorin (*DCN*)	F: CTAGTCACAGAGCAGCACCTAC	NM 001920.4
	R: CCAGGGAACCTTTTAATCCGGGAA	
Collagen type I (*COL1A1*)	F: CCCCAGCCACAAAGAGTCTAC	NM 000088.3
	R: TTGGTGGGATGTCTTCGTCT	
Collagen type III (*COL3A1*)	F: CCTGAAGCTGATGGGGTCAA	NM 000090.3
	R: CAGTGTGTTTCGTGCAACCAT	

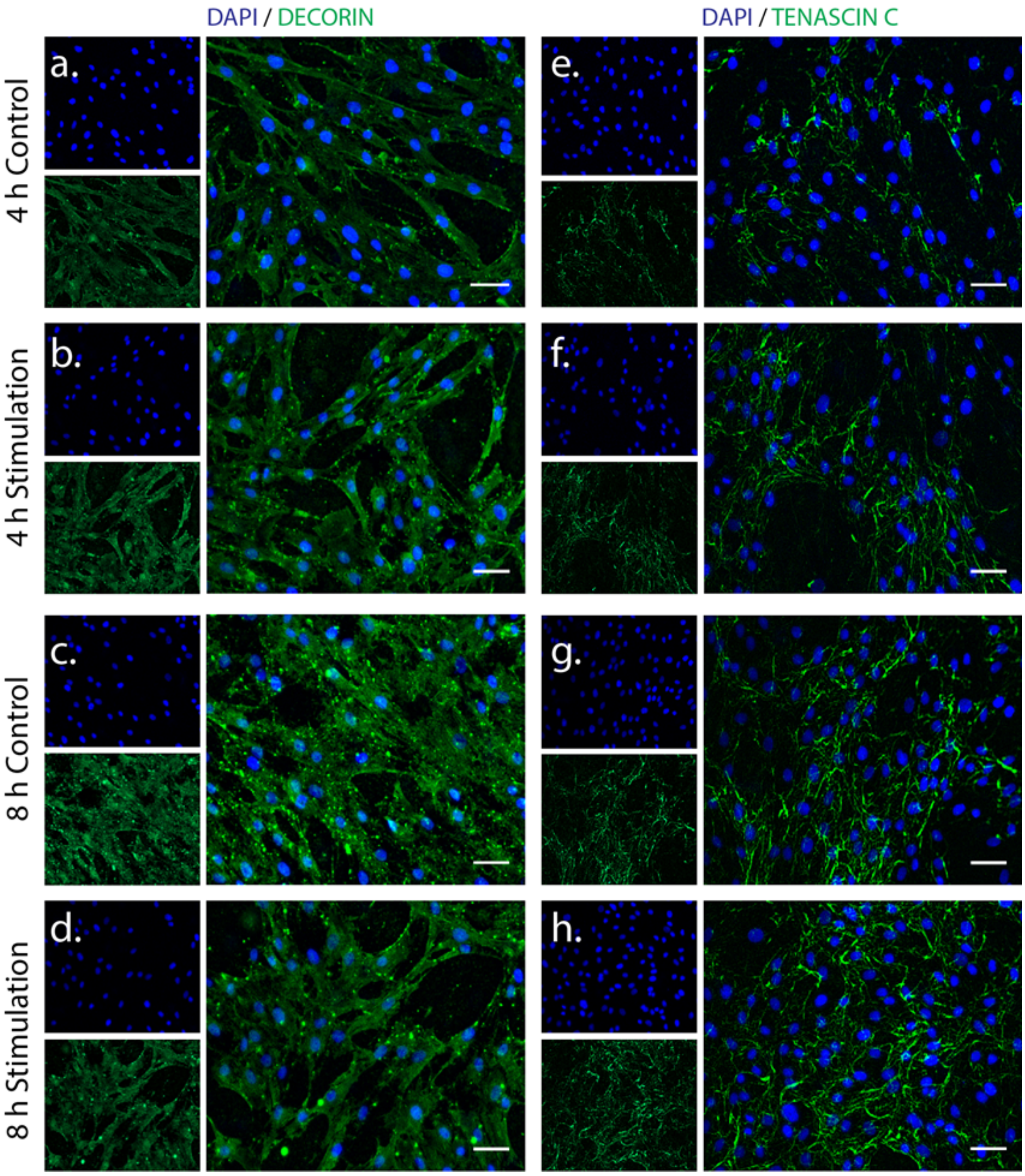

Supplementary Figure S1. Decorin and Tenascin C expression under short-term magnetic stimulation. (A-D) Fluorescence microscope images of immunostained decorin (green). (E-H) Fluorescence microscope images of immunostained tenascin c (green). Nuclei were counterstained with DAPI (blue). Scale bars, 50 μm.

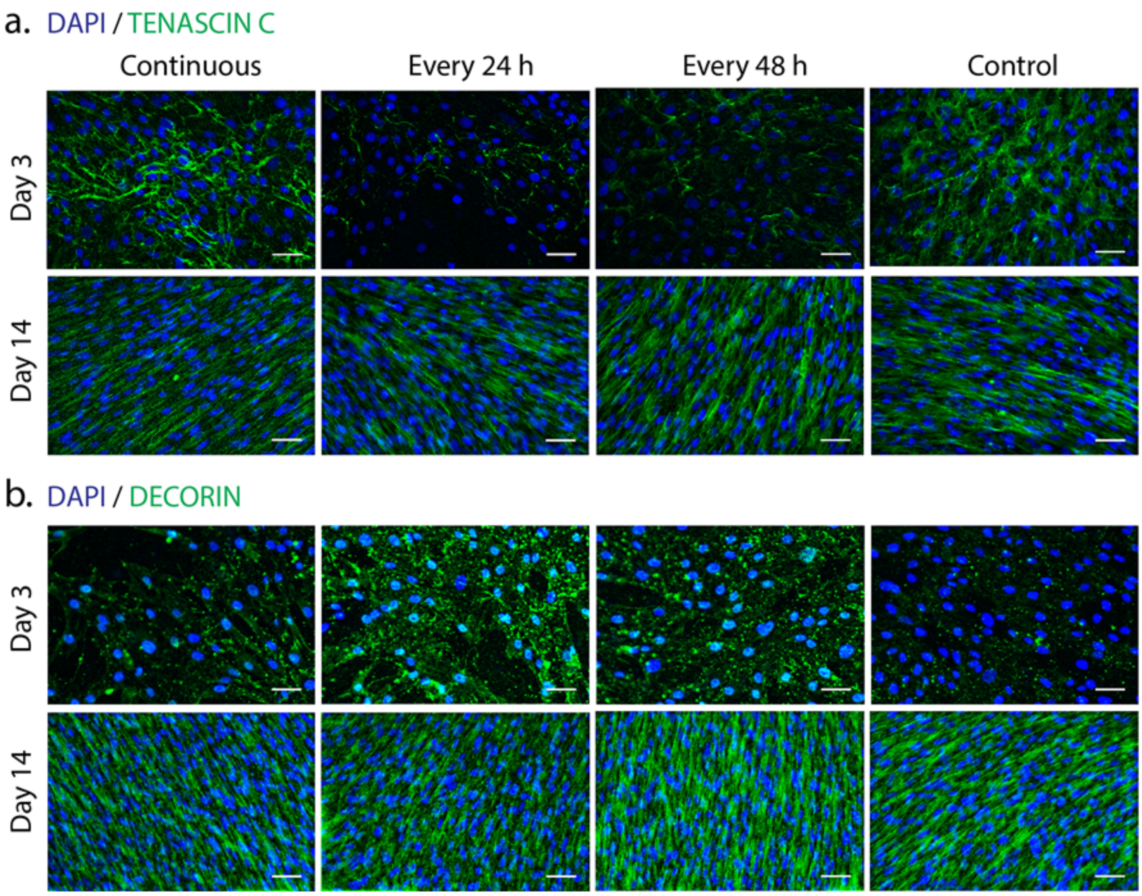

Supplementary Figure S2. Tenascin C and Decorin expression under long-term magnetic stimulation. (A) Fluorescence microscope images of immunostained tenascin c (green). (B) Fluorescence microscope images of immunostained decorin (green). Nuclei were counterstained with DAPI (blue). Scale bars, 50 µm.
